# Supplementary material for: Investigation of inherited noncoding genetic variation impacting the pharmacogenomics of childhood acute lymphoblastic leukemia treatment
Source: Nat Commun. 2024 May 1;15:3681. doi: 10.1038/s41467-024-48124-4 (PMC11063049; doi:10.1038/s41467-024-48124-4)
Supplement: Supplementary file 2 — Supplementary Information [file 41467_2024_48124_MOESM2_ESM.pdf]

SUPPLEMENTARY INFORMATION

Investigation of inherited noncoding genetic variation impacting the pharmacogenomics of childhood acute lymphoblastic leukemia treatment

Kashi Raj Bhattarai[1,2†], Robert J. Mobley[1,2†], Kelly R. Barnett, PhD[1,2], Daniel C. Ferguson[1,2], Baranda S. Hansen[3,4], Jonathan D. Diedrich[1,2], Brennan P. Bergeron[1,2,5], Satoshi Yoshimura[1,2,6], Wenjian Yang[1,2], Kristine R. Crews[1,2], Christopher S. Manring[7], Elias Jabbour[8], Elisabeth Paietta[9], Mark R. Litzow[10], Steven M. Kornblau[8], Wendy Stock[11], Hiroto Inaba[1,12], Sima Jeha[1,12], Ching-Hon Pui[1,12], Cheng Cheng[13], Shondra M. Pruett-Miller[3,4], Mary V. Relling[1,2], Jun J. Yang[1,2,5,14], William E. Evans[1,2] and Daniel Savic[1,2,5,14,*]

[1]Hematological Malignancies Program, St. Jude Children's Research Hospital, Memphis, TN 38105, USA
[2]Department of Pharmacy and Pharmaceutical Sciences, St. Jude Children's Research Hospital, Memphis, TN 38105, USA
[3]Center for Advanced Genome Engineering, St. Jude Children's Research Hospital, Memphis, TN 38105, USA
[4]Department of Cell and Molecular Biology, St. Jude Children's Research Hospital, Memphis, TN 38105, USA
[5]Graduate School of Biomedical Sciences, St. Jude Children's Research Hospital, Memphis, TN 38105, USA
[6]Department of Advanced Pediatric Medicine, Tohoku University School of Medicine, Tokyo, Japan
[7]Alliance Hematologic Malignancy Biorepository; Clara D. Bloomfield Center for Leukemia Outcomes Research, Columbus, OH 43210, USA
[8]Department of Leukemia, The University of Texas MD Anderson Cancer Center, Houston, TX USA
[9]Albert Einstein College of Medicine, New York, NY USA
[10]Division of Hematology, Department of Medicine, Mayo Clinic, Rochester, MN 55905, USA
[11]Comprehensive Cancer Center, University of Chicago Medicine, Chicago, IL USA
[12]Department of Oncology, St. Jude Children's Research Hospital, Memphis, TN 38105, USA
[13]Department of Biostatistics, St. Jude Children's Research Hospital, Memphis, TN 38105, USA
[14]Integrated Biomedical Sciences Program, University of Tennessee Health Science Center, Memphis, TN 38163 USA

[†]Authors contributed equally to this work
*Corresponding author: Daniel Savic, PhD
Division of Pharmaceutical Sciences
Department of Pharmacy and Pharmaceutical Sciences
St. Jude Children's Research Hospital
262 Danny Thomas Place
Memphis, TN, 38105
daniel.savic@stjude.org

SUPPLEMENTARY FIGURES

Supplementary Figure 1: ATAC-seq QC data. (a) TSS enrichment scores of the indicated categories of sample. **(b)** ATAC-seq narrowPeak counts of the indicated categories of sample. **(a-b)** For box and whisker plots the box encompasses the inner quartiles. The whiskers are the top and bottom quartiles. The line within the box represents the median value. Outlier points are defined as greater than 1.5 * IQR from the hinge (where IQR is the inter-quartile range). **(c)** The total number of peak summits of the indicated categories of samples is shown as a bar graph. The percentage of peak summits that were reproducibly identified in each category of samples is shown above.

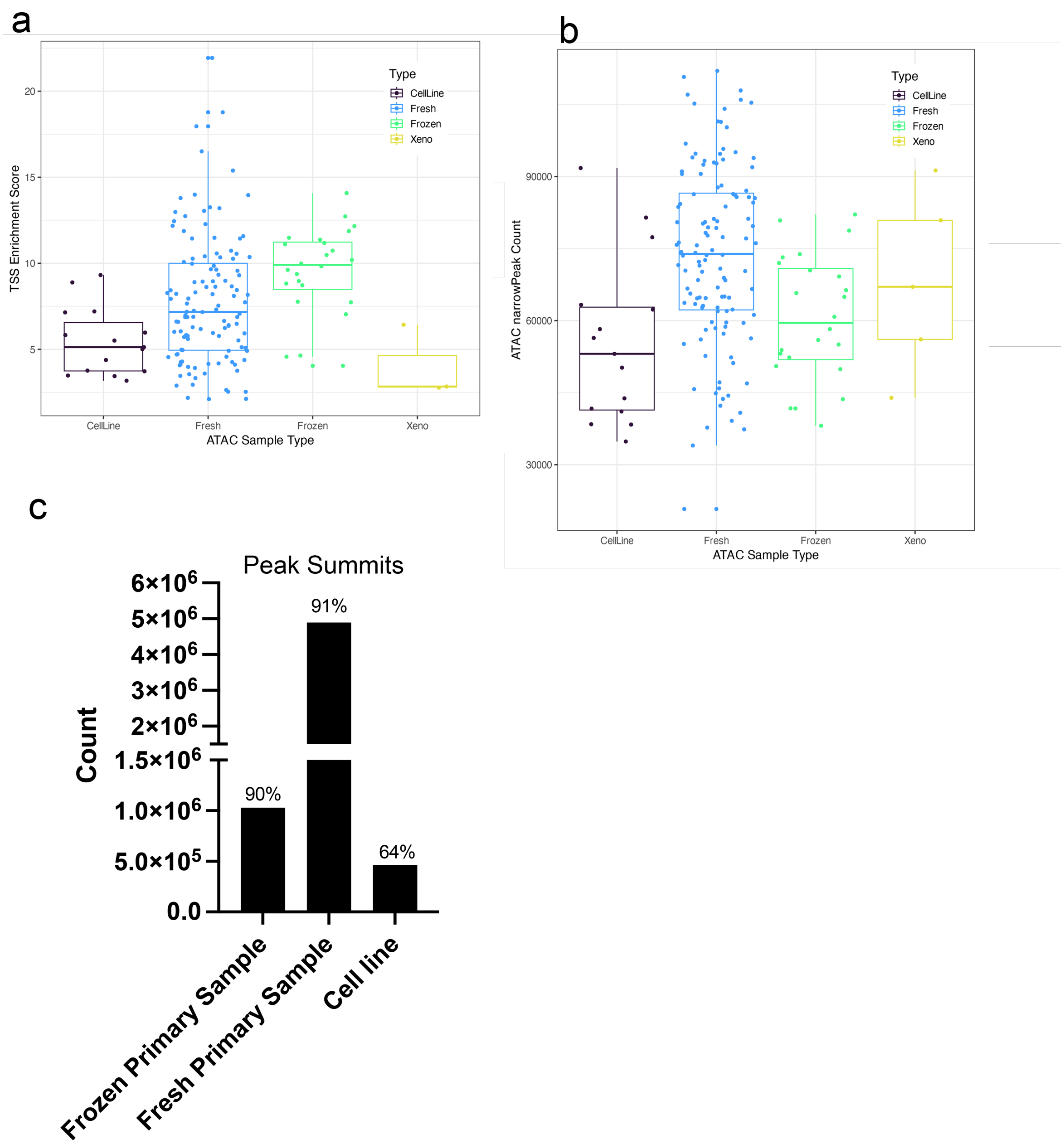

Supplementary Figure 2. MPRA activity comparisons. (a) Pairwise linear correlation between changes in allele-specific transcriptional activity for all measurements and across all cell lines. R^2 correlation and p-value are provided. **(b-c)** Plots showing the MPRA variant log fold change correlation between the two PDXs and ALL cell lines. For ALL cell line MPRA data, the mean log fold change is used. The linear regression +/- 95% CI is shown.

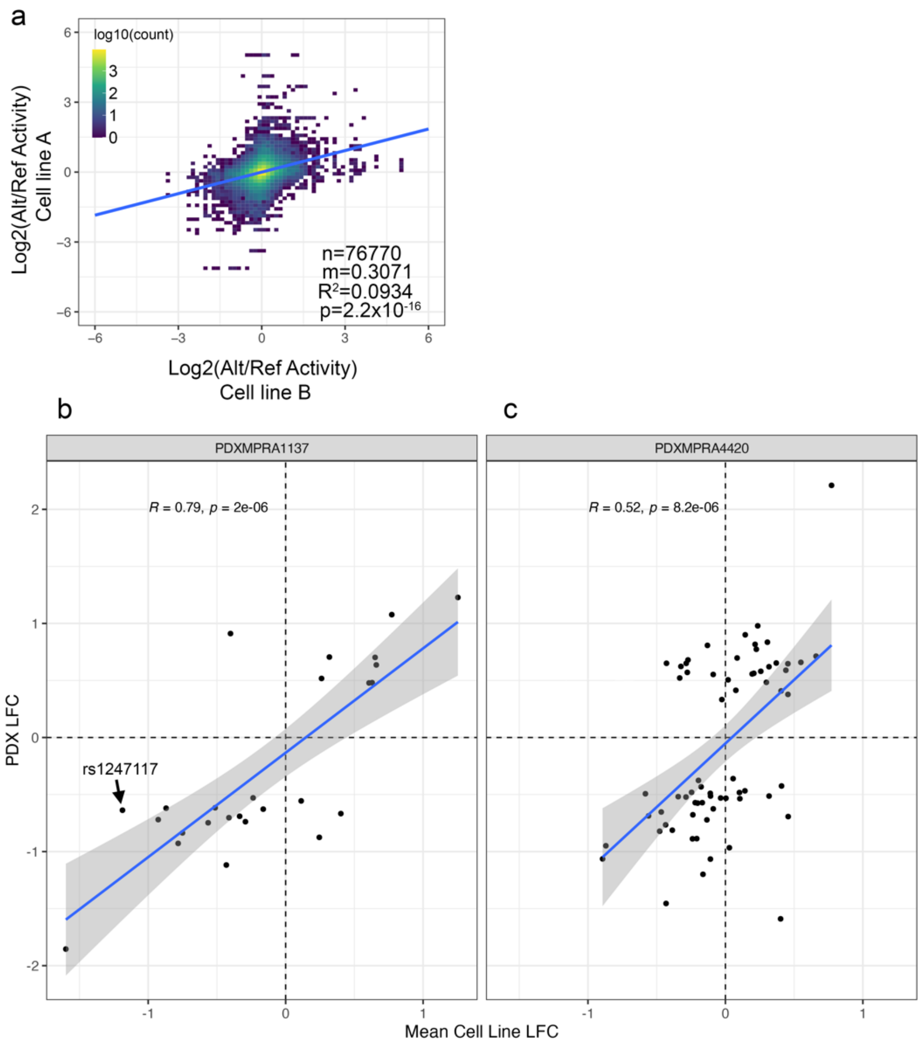

Supplementary Figure 3. Transcription factor footprints at functional regulatory variants. Transcription factor (TF) footprints identified at 54 of 556 functional regulatory variants are shown and ranked by the total number of motifs identified.

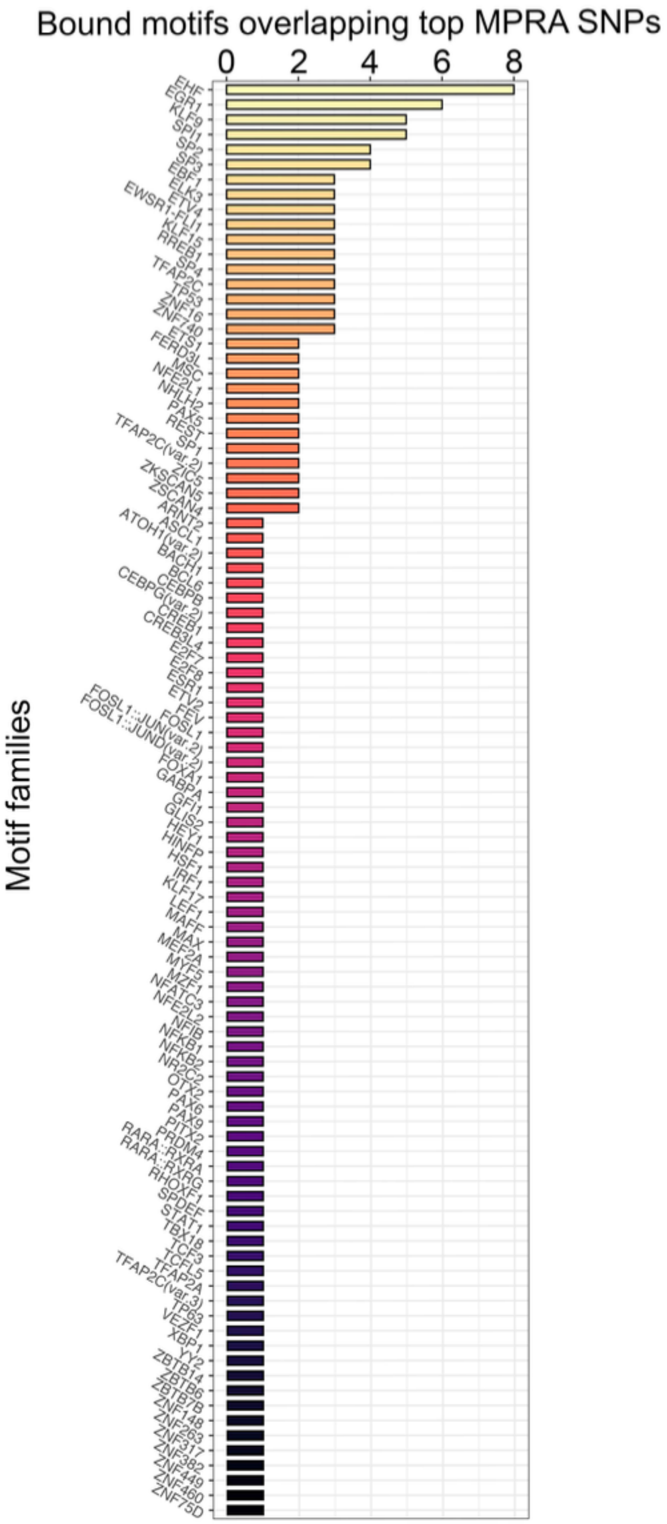

Supplementary Figure 4. Dual-luciferase reporter assay validation of the indicated functional regulatory variant. (a-k) Dual-luciferase reporter assays comparing the reference (Ref, in green) and alternate (Alt, in red) alleles ability to drive luciferase expression is depicted. Variant rs number and the ALL cell line the luciferase reporter assay was tested in is provided. (a) Data show the mean +/- SD of four independent experiments. P-value is calculated using a student's t-test. **(l)** Top hits from the 210 functional regulatory variants found in accessible chromatin in ALL cell lines were orthogonally validated by luciferase reporter assays. Data show significant correlation between the allele-specific effects detected by MPRA and dual-luciferase reporter assays.

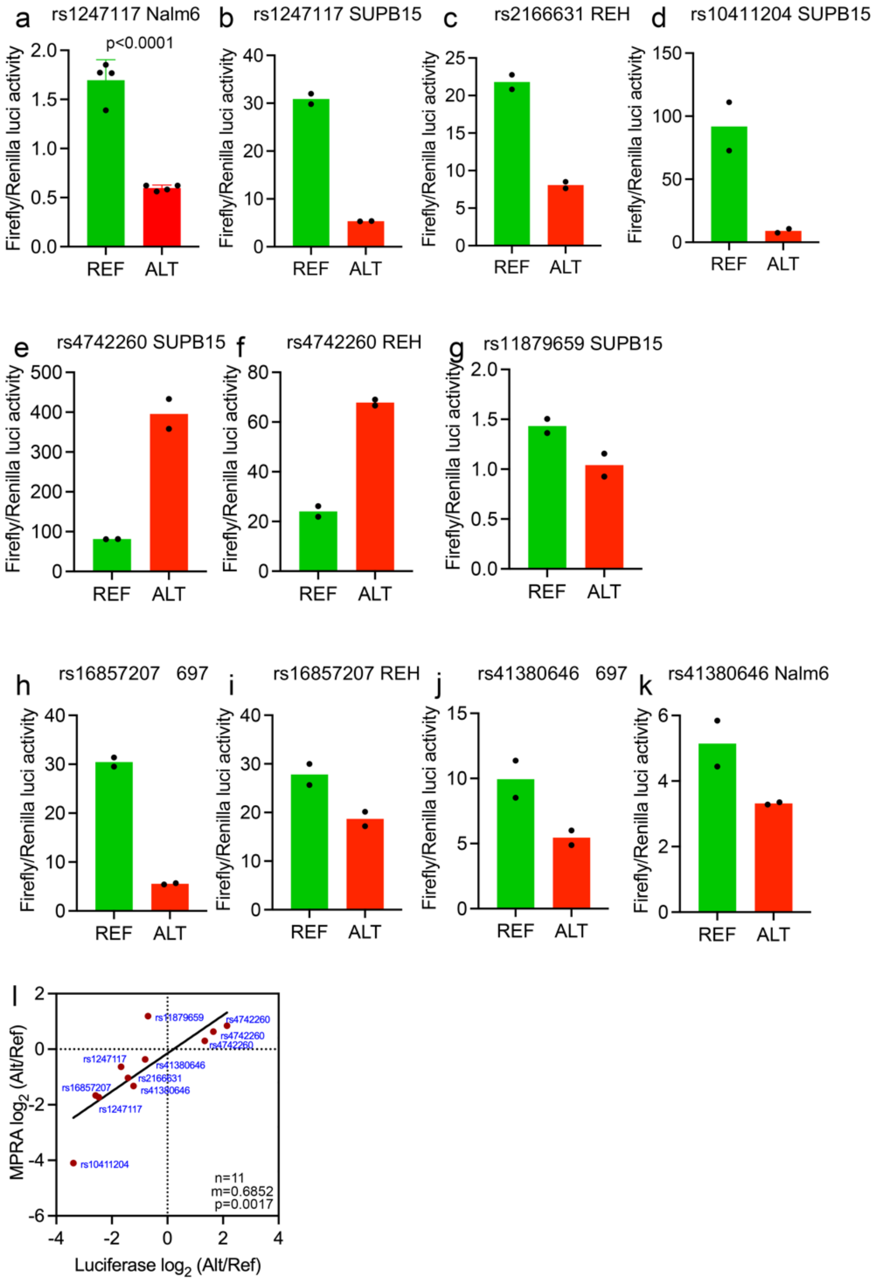

Supplementary Figure 5: H3K27Ac HiChIP versus Promoter Capture HiC. Bar graph shows significant loops called per cell line using each method. The total number of loops called is provided above the bar graph. Each bar represents one independent experiment.

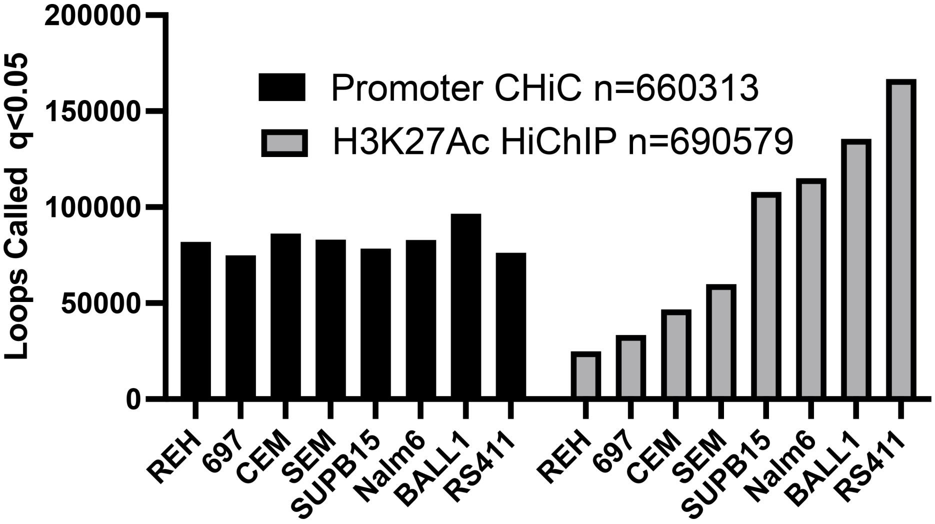

Supplementary Figure 6. Chromatin accessibility at rs1247117 in primary ALL cells. (a) IGV genome browser image of ATAC-seq chromatin accessibility spanning rs1247117 in diverse molecular subtypes of ALL is provided. **(b)** PU.1 footprint analysis comparing normalized ATAC-seq cut count signal for all bound PU.1 sites (red) compared to unbound (blue) sites across all primary ALL cells from patients. **(c)** Primary ALL cells with SNV genotype information were analyzed (n=69 samples). Normalized ATAC-seq read counts in heterozygous (GA) primary ALL cells (n=12 samples) at rs1247117 compared to homozygous (AA) primary ALL cells (n=57 samples). Mann Whitney U test p-value is provided. **(d)** Normalized ATAC-seq read counts per allele in primary ALL cells for G allele (n=12 samples) and A allele (n=69 samples). Normalized counts for G and A alleles are shown. Mann Whitney U test p-value is provided.

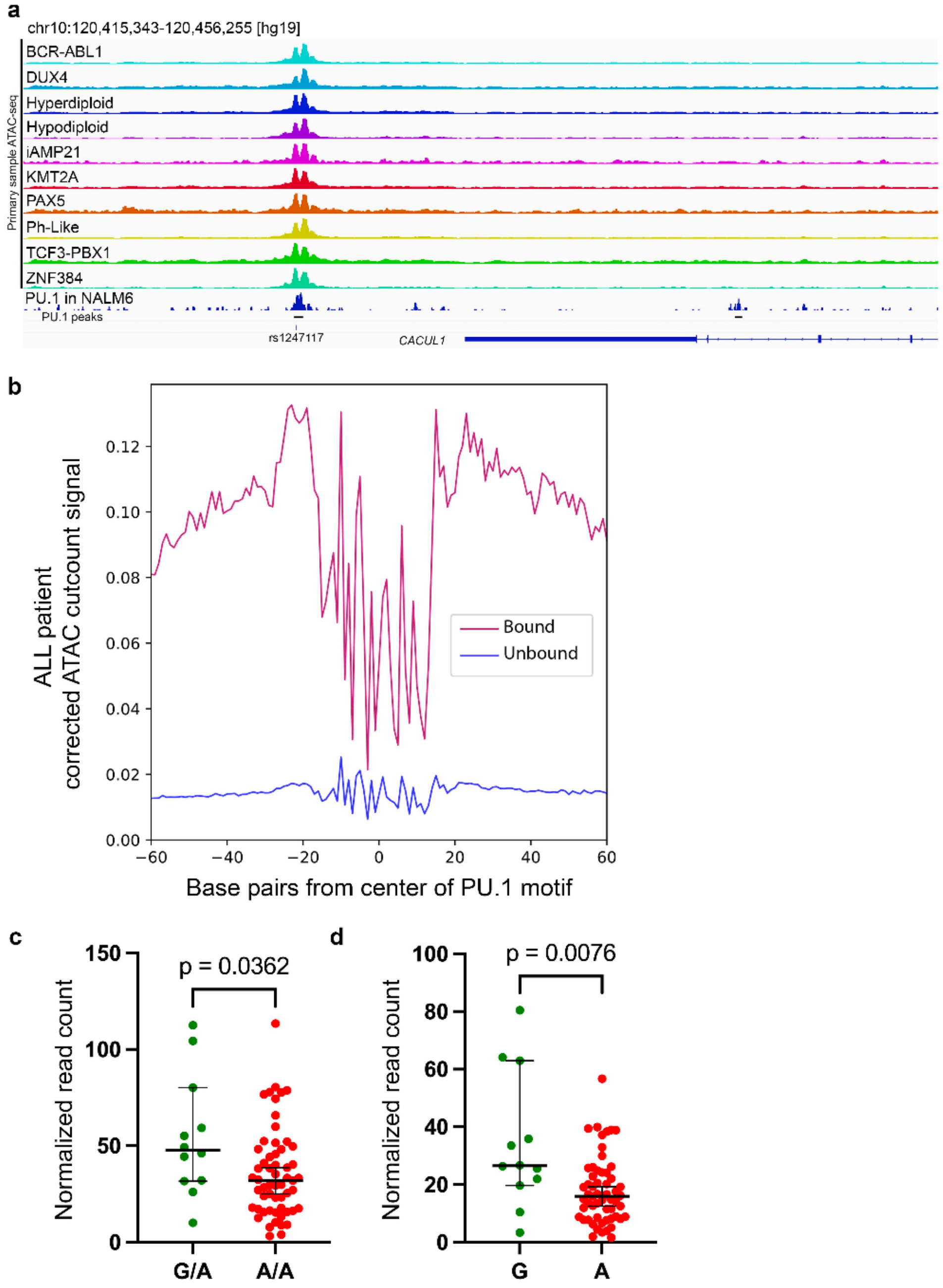

Supplemental Figure 7: rs1247117 CRE deletion in SUPB15 cells leads to increased EIF3A expression and increased sensitivity to vincristine. (a) CRISPR/Cas9 was used to delete the ~1kb CRE region containing rs1247117. Unmodified cells yield a fragment of ~1300bp whereas the modified pools predominantly yield a ~300bp fragment of this region. **(b)** Western blots and quantification showing elevated expression of EIF3A in SUPB15 cells. Images shown are representative of 3 independent experiments. A two-tailed Students T-test p-value is provided. **(c)** Drug sensitivity data comparing survival of wild-type parental SUPB15 cells and SUPB15 cells with rs1247117 CRE deletion after vincristine (VCR) treatment for 72 hours (n=3 independent experiments). Dose-response curves of non-linear regression indicate that these curves are significantly different.

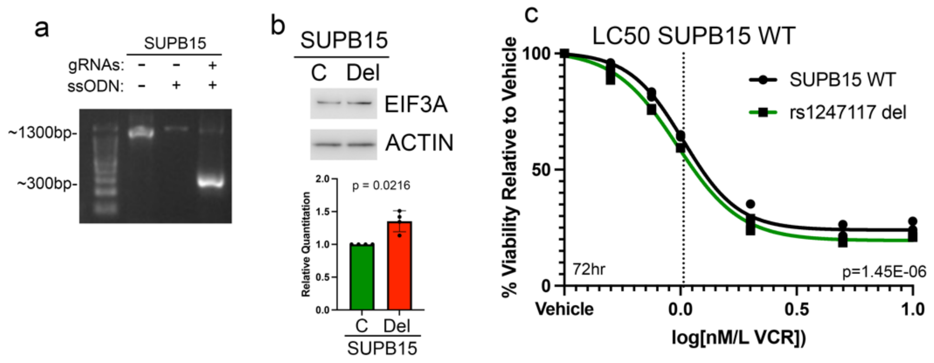

Supplementary Figure 8: rs7426865 CRE deletion impacts 6-mercaptopurine sensitivity in SUPB15 cells. (a) Genomic locus showing rs7426865 with promoter capture Hi-C data and ATAC-seq data from SUPB15 cells. **(b)** CRISPR/Cas9 was used to delete the CRE containing rs7426865 in SUPB15 cells. Data shown are next-generation sequencing reads from edited cell pools. **(c)** Western blots of whole cell lysate from the indicated cells for the indicated putative target genes of the rs7426865 CRE. Blots shown are representative of 2 independent experiments. **(d)** rs7426865 CRE deletion in SUPB15 cells leads to resistance to 6-mercaptopurine (6MP) after 72hrs of treatment at the indicated doses (n=6 technical replicates from two independent experiments). Dose-response curves of non-linear regression indicate that these curves are significantly different (p<0.0001).

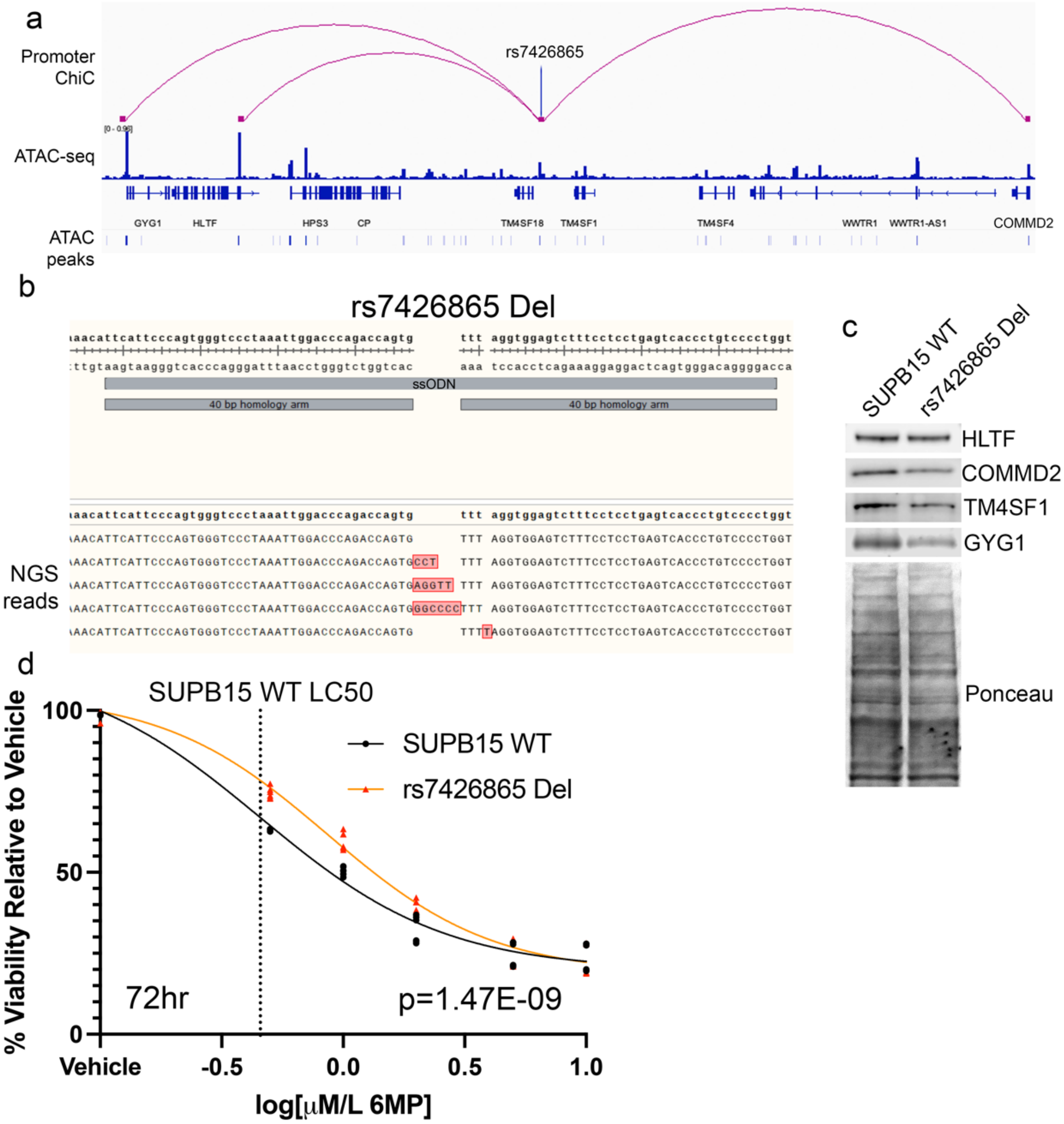

Supplementary Figure 9: rs12660691 CRE deletion impacts dexamethasone sensitivity in SUPB15 cells. (a) Genomic locus showing rs12660691 with promoter capture Hi-C data and ATAC-seq data from SUPB15 cells. **(b)** CRISPR/Cas9 was used to delete the CRE containing rs12660691 in SUPB15 cells. Next-generation sequencing reads showing deletion of the CRE containing rs12660691. **(c)** Western blot showing ARHGAP18 protein levels in WT and rs12660691 CRE deletion SUPB15 cells. Data shown are two independent experiments. **(d-f)** rs12660691 CRE deletion in SUPB15 cells leads to resistance to dexamethasone after 24, 48 and 72hrs of treatment at the indicated concentrations (n=3 independent experiments). Dose-response curves of non-linear regression indicate that these curves are significantly different.

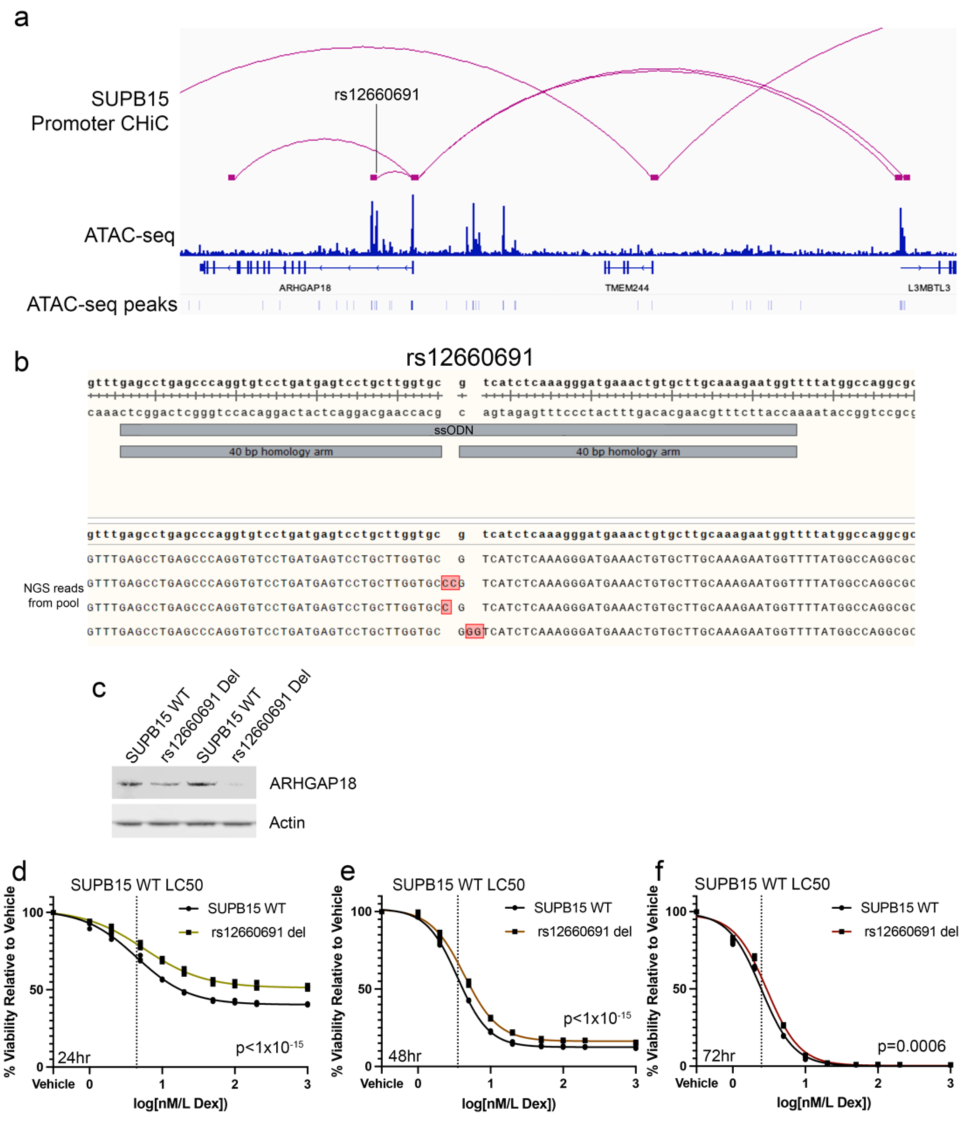

Supplemental Figure 10: EIF3A overexpression. (a) Western blots and quantitation showing elevation of EIF3A protein after two independent lentiviral transductions with an EIF3A overexpression construct (EIF3A OE I1 and EIF3A OE I2) or Nalm6 cells infected with a control construct. Images shown are representative of two independent experiments. A two-tailed Students T-test p-value is provided. **(b)** Drug sensitivity data comparing survival of control-infected Nalm6 cells and Nalm6 cells with EIF3A overexpression after vincristine (VCR) treatment for 48 (n=3 independent experiments) or 72 (n=3 independent experiments) hours at the indicated concentrations. Dose-response curves of non-linear regression indicate that these curves are significantly different.

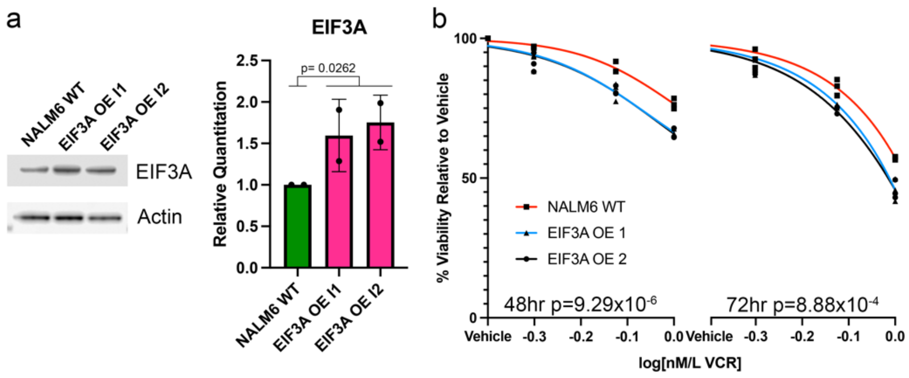

Supplemental Figure 7b source data

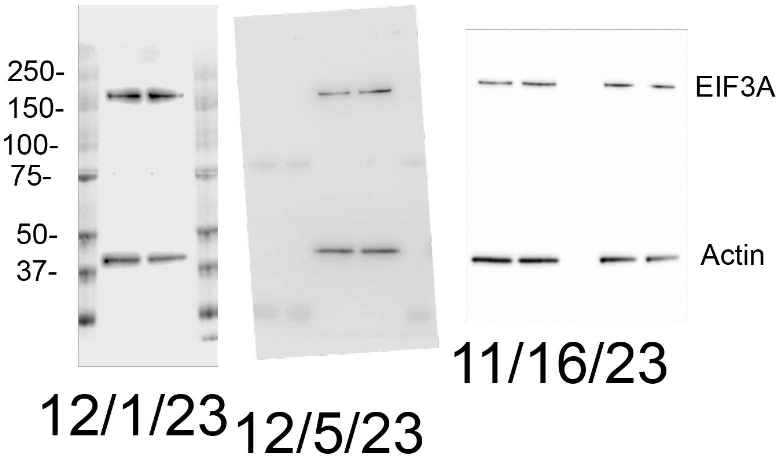

Supplemental Figure 8c source data

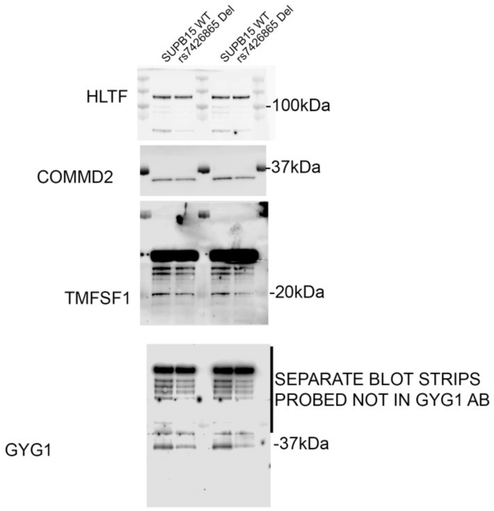

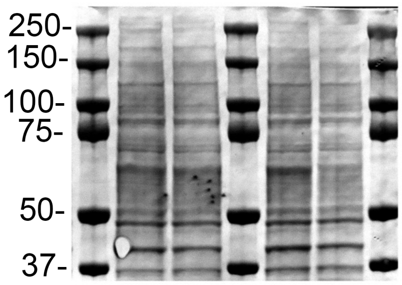

Supplemental Figure 9c source data

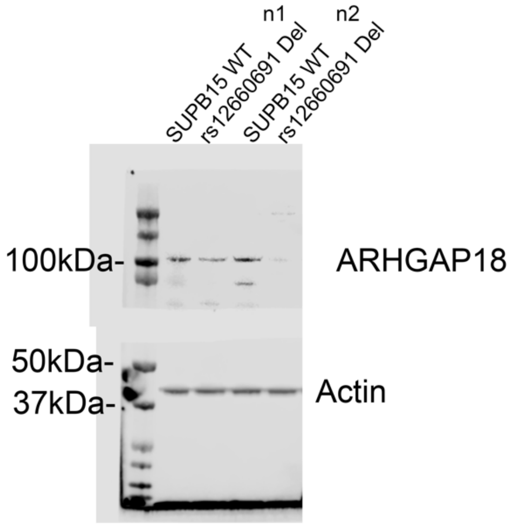

Supplemental Fig 10a source data

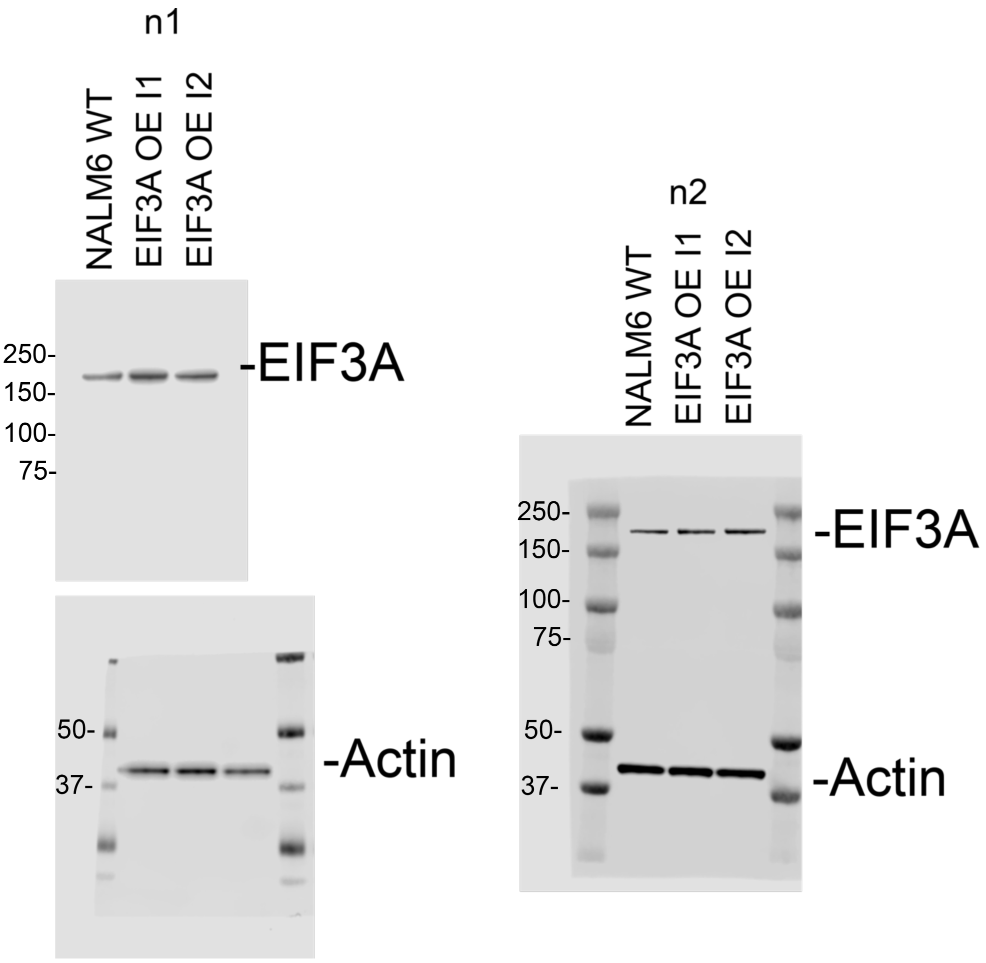

SUPPLEMENTARY METHODS

Dual-luciferase reporter assays

A 300-bp of sequence centered on reference or the alternative allele of rs1247117, rs10411204, rs4742260, rs12660691, rs2166631, rs11879659, rs41380646, rs16857207 was cloned upstream of the minimal promoter into the pGL4.23-basic vector. Sequences used in luciferase reporter experiments are shown in **Supplementary Data 8**. Nalm6, SUPB15, REH, and 697 cells (10 million cells per replicate, 60 µg plasmid DNA and 6 µg pRL-TK control vector) were used for transfection. Using Neon Transfection system (Thermo Fisher Scientific, MPK5000), the constructs were co-transfected with renilla plasmid to enable normalization of the luciferase signal. 24 h post-transfection, firefly luciferase and renilla luciferase activity was measured using Dual Luciferase Reporter Assay System (Promega, E1960) on a BioTek Cytation1 cell imaging multimode reader (Agilent). The ratio of firefly luciferase to renilla luciferase activity readings reflect the luciferase activity of the reference allele relative to the alternative allele. All experiments were performed in 10 samples from each replicate and repeated 2 or 4 times.

Massively parallel reporter assays

MPRA Oligo design

Oligo libraries were designed by following previous work with modified protocols [1-4]. MPRA oligos ordered from Agilent (230 bp) were structured as follows: 5’-Primer1-enh-KpnI-XbaI-barcode-primer2-3’ where primer1 and primer2 are universal primer sites, *enh* denotes the 175bp variant containing region to test for enhancer activity, KpnI and XbaI denote recognition sequences for cut sites, and *barcode* denotes 10-bp tag sequence (see **Supplementary Data 9**). Agilent oligos were resuspended in 100 ul nuclease free water. All 10-bp barcodes for each variant allele used in MPRA are provided in **Supplementary Data 9**.

MPRA Plasmid Cloning-Input (DNA) Library construction

For plasmid cloning, oligo libraries were amplified by 20 cycles of emulsion PCR (Micellula DNA Emulsion & Purification Kit #E3600, EURx Molecular Biology Products) using Herculase II fusion DNA polymerase (#600675, Agilent), forward and reverse primers (see Sup File 3) to introduce SfiI restriction enzyme sites (GGCCNNNNNGGCC) (NEB) and homology arms to the pMPRA1 plasmid (Cat: #49349, Addgene). Purified PCR products were separated on a 2-4% agarose gel to verify the expected amplification size of 281bp. The pMPRA1 backbone vector was SfiI digested overnight and size selected on a 1% agarose gel. Vector backbone gel extraction was done with the Qiagen gel extraction kit and QIAquick PCR purification kit (28706X4 and 28104). Gibson assembly was used to clone oligos into vector using 79 ng of inserts, 100 ng of digested vector, and 20 ul Gibson assembly 2x master mix (# E2611S, NEB). The reaction was purified using MinElute PCR purification column (Qiagen), and drop analysis was performed with Millipore filters (Type VSWP 0.25 um Millipore #VSWP02500). For the transformation step, we aimed to obtain 10x CFU bacterial cells than the distinct promoter-tag combinations (unique sequences) in the oligo library. We transformed the Gibson assembly reactions into MegaX DH10B electrocompetent bacteria (#C6400-03, Invitrogen) using GenePulser II electroporator (Bio-Rad). Plasmids were extracted with Qiagen Maxi Prep Kit. For quality control studies, an aliquot of the isolated plasmid library was digested with SfiI and run on 1 % agarose gel to confirm the presence of inserts. To generate linear enhancer-barcode backbone sequences for reporter insertion, 2 ug of plasmid was digested with KpnI/XbaI. A minimal promoter + truncated *eGFP* was then ligated to the linearized enhancer-barcode backbone and purified using the Qiagen MilEute PCR purification kit. The ligation was then transformed into 1 vial of MegaX electrocompetent bacteria (#C6400-03, Invitrogen)

as before. Plasmids were then extracted using a Qiagen Maxiprep kit as before and the elution was verified as a single size band by gel electrophoresis.

MPRA library transfection and sequencing

MPRA plasmid library (10 µg/10M cells) transfections were done in 10 ALL cell lines having at >95% cell viability (45 million cells x 4 replicates x 10 cell lines) using electroporation with the Neon Transfection system (Thermofisher, see **Supplementary Data 10**). Next day, RNA-was harvested using the RNeasy plus mini kit (Cat: #74134, Qiagen) using 4 columns per sample. Once the RNA was isolated, we performed additional DNase digestion using RQ1 RNase-free DNase (Cat: # M6101, Promega). All tubes from the same replicates were combined and added 1 volume of 70% ethanol to the combined lysate and mixed well by pipetting. The DNase treated RNA was again purified with RNeasy mini kit and eluted in 60 ul RNase free water. Total RNA was quantified using DeNovix Ds-11 FX instrument. We yielded 16µg-112µg of total RNA from each replicate depending on the cell line used. mRNA purification was performed using Dynabeads mRNA purification kit (Cat: #61006, Invitrogen). mRNA concentration was measured using Qubit HS RNA (Cat: #Q32852, Invitrogen). We yielded from 0.75-2 ug of mRNA in average from each replicate. cDNA was synthesized using three primers (2 uM) cDNA P1, cDNA P4, and cDNA 6, with the Superscript III first-strand synthesis system (Cat: #18080051, Invitrogen) (see **Supplementary Data 11**).

Final multiplexing of 50ng cDNA and input plasmid DNA (4 aliquots of MPRA plasmid pool that were independently prepared for next-generation sequencing) was carried out using Q5 Hot Start 2x Master Mix (NEB #M0494S), index primers, and Multiplexing primer 1 for 15 cycles of PCR. The reactions were size selected using AMPure XP beads (Cat: #A63881, Beckman Coulter, Indianapolis, IN) and eluted in 20 ul nuclease free water. The final library concentration was measured using Qubit DNA HS.

20-40 ng of each library was sequenced on the Illumina NovaSeq (200 million x 150bp paired-end reads per sample) at the Hartwell Center for Bioinformatics and Biotechnology at St. Jude Children's Research Hospital.

Patient derived Xenograft (PDX) MPRA and transfection

To test patient-derived xenograft (PDX) samples, 2 pediatric B-ALL PDX samples were obtained from Children's Hospital of Philadelphia and expanded using NOD.Cg-*Prkdc*^{scid}*Il2rg*^{tm1Wjl}/SzJ (NSG) mice from Animal Resource Center at St. Jude. One to two million human B-ALL cells per mouse were injected via the tail vein into female NSG mice aged between 8 and 12 weeks. All NSG mice were housed in sterilized conditions at 20–23 °C and 40–60% humidity, with a 12 hrs light–12 hrs dark cycle applied and their health statuses were monitored daily. Peripheral blood was collected biweekly to monitor the burden of human leukemia by flow cytometry: cells were stained with mTER119 (BioLegend, #116228; diluted at 1:200), mCD45 (BD Sciences, #557659; 1:100), hCD45 (BD Sciences, #555482; 1:50) and hCD19 (BioLegend, # 363004; 1:100); hCD45 and hCD19 double-positive percentage was determined using a BD FACS LSR II machine (BD FACS Diva Software v.9). Mice were euthanized when leukemia cells reached 80% in peripheral blood, or they became moribund. Human leukemia cells were collected from spleen and enriched using an immunomagnetic isolation kit (Stemcell Technologies, #19849) for further studies. This study was approved by the respective institutional review boards and all the animal studies were approved by the Institutional Animal Care and Use Committee of St. Jude.

For PDX cells, MPRA transfection was done in 50-90 million cells/replicate and using 4 replicates per sample. After an overnight incubation for 24hr the RNA-was harvested using the RNeasy Midi Kit (Cat: #75144, Qiagen). PDX cells were transfected as shown in **Supplementary Data 10.**

MPRA sequencing analysis

Following next-generation sequencing, the MPRA sequence data was trimmed to contain only barcode sequences without allowing for any mismatches and read counts were determined for all barcodes. To identify significant allele-specific effects mpralm [5] was performed on RNA and DNA barcode counts after merging RNA or DNA counts across all barcodes for each allele.

Quantitative real time PCR (qPCR)

Nalm6 parental (WT) and *rs1247117* deleted Nalm6 cells were cultured in RPMI 1640 media + 10% serum. 10 million cells were collected from each group in triplicates and resuspended in RLT/BME mixture for total RNA extraction. RNA was isolated using RNeasy Mini Kit (Qiagen #74104). Complimentary DNA synthesis was done using the High-Capacity RNA-to-cDNA Kit (Applied Biosystems #4387406). RT-PCR reactions were prepared using TaqMan Fast Advanced Master Mix (Applied Biosystems #4444557) and TaqMan Gene Expression Assays (Thermo) (*EIF3A*: Hs01025769_m1, *TBP* endogenous control, Hs00427620_m1). The samples were run on a QuantStudio 3 Real-Time PCR Instrument using the recommended TaqMan Fast Advanced Master Mix PCR conditions.

Chromatin accessibility mapping in ALL cell models

Fast-ATAC in fresh primary ALL cells from patients (n=120), PDXs (n=3) and in a subset of ALL cell lines (BALL1, CEM, Jurkat and P12-Ichikawa) was performed on 10,000 cells as described in [6,7]. Briefly, 10,000 cells were washed with PBS and suspended in tagmentation master mix including 0.01% digitonin and incubated at 37°C according to the manufacturer's instructions (Illumina Tagment Enzyme and Buffer Kit #20034198). Library prep of tagmented DNA was performed using NEBNext® Ultra™ II Q5® Master Mix (M0554) and nextera compatible primers

(IDT). Paired-end Illumina next-generation sequencing of Fast-ATAC libraries was performed at the Hartwell Center for Bioinformatics and Biotechnology at St. Jude Children's Research Hospital. Data were analyzed as in. For cryo-preserved primary ALL cells, fresh primary ALL cells and B-ALL cell lines, Fast-ATAC data was obtained from the Gene Expression Omnibus (GSE129066, GSE161501 and GSE211631) [8,9].

Promoter capture Hi-C

Arima promoter capture HiC (Arima: A510008, A303010, A302010) was performed according to the manufacturers provided instructions using unspecified proprietary buffers, solutions, enzymes, and reagents. Briefly, 10 million ALL cells were harvested, suspended in 5ml RT PBS which was brought to 2% formaldehyde by adding 37% methanol-stabilized paraformaldehyde for a 10-minute fixation. The amount of fixed cell suspension equal to 5μg of cell DNA was used for HiC. Cells were lysed with Lysis Buffer and conditioned with Conditioning Solution before their DNA was digested in a cocktail consisting of Buffer A, Enzyme 1, and Enzyme 2. The digested, fixed chromatin was biotinylated using Buffer B and Enzyme B before being ligated using Buffer C and Enzyme C. The fixed, biotinylated, ligated DNA was then subjected to reversal of crosslinking and digestion of proteins before being purified. 100ul containing 1500ug of purified large proximally ligated DNA was fragmented for 24 cycles (30s on/ 30s off) using a Diagenode Bioruptor Plus bath sonicator. The fragmented DNA was then subjected to two-sided size selection targeting fragments between 200-600bp using AMPure XP DNA purification beads. Size selected DNA was then subjected to biotin enrichment using T1 streptavidin beads. Bead bound, enriched HiC DNA was then subjected to Arima library prep. Briefly, the sample underwent end repair followed by adapter ligation, at which point the sample was then subjected to 10 cycles of PCR amplification. The library DNA was then purified using AMPure XP DNA purification beads. The HiC library was then subjected to Arima promoter capture enrichment. The library was precleared of biotinylated DNA using T1 streptavidin beads before being subjected to promoter

enrichment with biotinylated RNA probes. After washing, the captured fragments were then amplified an additional 13 PCR cycles. These libraries were submitted for deep sequencing on an Illumina Nova-seq where >200M 150bp paired-end reads were obtained. Analysis of Promoter capture HiC data was performed using the Arima CHiC pipeline (v1.5, https://github.com/ArimaGenomics/CHiC). Briefly, this pipeline uses HiCUP v0.8.0 for mapping and quality assessment of promoter capture HiC data and CHiCAGO to identify significant looping interactions in the promoter capture HiC data using 5kb resolution and adj. $p < 0.05$ [10,11].

H3K27Ac HiChIP

Arima H3K27Ac HiChIP (Arima: A101020) was performed according to the manufacturers provided instructions using unspecified proprietary buffers, solutions, enzymes, and reagents. Briefly, cells were fixed as in promoter capture Hi-C above and the amount of fixed cell suspension equal to 15μg of cell DNA was used for the HiC reactions as in promoter capture Hi-C above. The samples were then sonicated 25 cycles (30s on/ 30s off) using a Diagenode Bioruptor Plus bath sonicator. Chromatin immunoprecipitation of the sonicated HiC samples was performed by first incubating the samples with 0.2ug of H3K27Ac antibody (Active Motif 91194) per 1ug of sample overnight. The next day antibody bound chromatin was collected using protein A beads included in the kit. The beads were washed three times with buffer R1, twice with buffer R3, once with buffer LC and then twice with LTE (buffers specified in Arima kit). Samples were eluted before undergoing decrosslinking. Libraries were prepared using Swift Biosciences Accel-NGS 2S Plus DNA Library Kit (Cat # or 21096) following instructions provided by Arima Genomics. The paired-end libraries were sequenced on the Illumina Novaseq to generate >200M 150bp reads. The provided FitHiChIP pipeline was used for analysis to call 5kb resolution loops in relaxed background mode (https://github.com/ay-lab/FitHiChIP)

PU.1 ChIP-qPCR

For PU.1 ChIP-qPCR, ChIP was performed using 4M cells per sample fixed for 10' in 1% formaldehyde. Samples were sonicated 25 cycles (30s on/ 30s off) using a Diagenode Bioruptor Plus bath sonicator. Before antibody addition, a 1% input sample was taken. Chromatin immunoprecipitation of the sonicated samples was performed by first incubating the samples with 1ul of PU.1 antibody (Cell Signaling 2258) per sample overnight. The next day antibody bound chromatin was collected using protein A beads. The beads were washed three times with buffer R1 (10mM Tris pH8, 140mM NaCl, 1mM EDTA, 1% Triton X-100, 0.1% SDS, 0.1% sodium deoxycholate, 1% protease inhibitor cocktail (Sigma P8340-5ML)) twice with buffer R3 10mM Tris pH8, 150mM LiCl, 1mM EDTA, 0.5% IGEPAL CO-630, 0.1% sodium deoxycholate), once with buffer LC and then twice with LTE (10mM Tris pH8, 0.1mM EDTA). Samples were eluted during decrosslinking in SDS/proteinase K at 65C alongside the input samples. ChIP and input DNA was purified using AMPure XP DNA purification beads before being subjected to qPCR. We used three sets of PCR primers (IDT) with amplicons <120 bp to amplify DNA from Nalm6 cells with either the A or G allele at *rs1247117* using the PowerTrack SYBR Green Master Mix (Thermo Fisher Scientific, see **Supplementary Data 12** for primers). PU.1 signal was quantified relative to the input DNA signal.

PU.1 *in vitro* binding affinity assay

DNA pulldown assay was adopted from previous article and performed using a modified protocol [12]. Briefly, biotinylated ssDNA probes were ordered via custom synthesis from IDT with their non-biotinylated reverse complement sequences. The DNA probe sequences used in the experiment are listed in **Supplementary Data 13**. The probes containing reference and alternative alleles featuring the nucleotide of interest (*rs1247117*) and its flanking +/-12 bp nucleotides were arranged side-by-side in tandem for a total of 50 bp each. Probes were annealed by combining biotinylated probes and the non-biotinylated reverse complement at 1.5M excess (50µM:75µM)

with an equal volume of 2x annealing buffer (10 mM Tris pH 8.0, 100 mM NaCl, 2 mM EDTA) and incubating at 98C for 10 minutes before cooling at RT overnight. To isolate nuclear lysate, we washed 75 million cells in 5 mL PBS and pelleted at 500xg for 3 min at RT. The cells were resuspended in 2 mL of homogenization buffer (1M KCl, 1M MgCl2, 1M HEPES, 0.5 M EGTA, 1x Halt protease inhibitors, Thermo Fisher 78429) and passed through 26 G needle 10x. The nuclei were pelleted, and the supernatant was discarded. The nuclei were gently washed with an additional 1ml of homogenization buffer, pelleted, and the supernatant discarded again. Washed nuclei were suspended in 300 µL of SKT buffer (1M HEPES, 1M MgCl2, Glycerol, 1 M KCl, EDTA, 0.1% triton). To extract nuclear proteins 33ul of 3M NaCl was added and samples were vortexed every 2-3 min on ice for the next 20 min. Insoluble nuclear material was pelleted at 13000 rpm for 10 min at 4°C. To make a nuclear lysate master mix 1100µg of nuclear lysate was transferred to a fresh tube with 22µg nonspecific DNA (11µg Poly (dI-dC), (Thermo Fisher 20148E) + 11µg Poly(dA:dT), (Cell Signaling Technologies)) and brought to a final volume of 1320µL with protein binding buffer (PBB, 150 mM NaCl, 0.25% NP40, 50 mM Tris pH 8.0, 1 mM DTT and EDTA free protease inhibitors (Roche). Reference and Alternative allele annealed biotinylated DNA probes (500pMol) were prebound to 20ul Streptavidin T1 Dynabeads (#65601, Thermo) in 600µL DNA binding buffer (DBB, 1 M NaCl, 0.05% NP40, 10 mM TRIS, pH 8.0 and 1 mM ETDA) rotating at 4°C for 30 min. Streptavidin T1 beads bound to probes were washed 1x DBB, 2x PBB on ice and 600 uL of nuclear lysate master mix (prepared above) was added to reference or alternative allele bead-bound probe tubes. Lysate and probe-bound beads were rotated for 90 min at 4°C and washed 3x PBB, and 2x PBS by pipetting up and down 5x each wash. Proteins were eluted in 40 ul 1x LDS sample buffer (+10% BME) (Cat: #NP0007, Invitrogen) by heating at 99.9°C in a thermal mixer at 1200 rpm for 10 min (#13687712, Thermo). Samples were subsequently assessed as described in **Western blotting** using anti-rabbit PU.1 antibody (#2258S, Cell Signaling Technology).

ChIP-seq

RNA polymerase II ChIP-seq data were generated by first fixing 20 million Nalm6 cells in 1% formaldehyde (diluted from sigma F87750) at room temp for 10 minutes. Crosslinking was stopped with the addition of 2.5M glycine to a concentration of 0.125M, and the cells were then washed in ice-cold PBS. 5μg anti-RNA polymerase II CTD repeat YSPTSPS (phospho S5) antibody [4H8] (ab5408, lot: GR3264797-1) was prebound to 200ul of protein G dynabeads (Invitrogen 10003D) overnight in 0.5% BSA in PBS. Fixed cell pellets (20M cells) were suspended in 1ml Farnham lysis buffer (5mM PIPES pH 8, 85mM KCl, 0.5% NP40, 1x protease inhibitors (Roche 11836170001) and passed through a 18G needle 10x. Nuclei were pelleted and resuspended in 275ul of RIPA buffer (1x PBS, 1% NP40, 0.5% Sodium Deoxycholate, 0.1% SDS, 1x protease inhibitors) and sonicated on high power in 1.5ml tubes for 25 minutes (30s on/ 30s off) using a Diagenode Bioruptor Plus. 5% Input samples were taken from sonicated material and the remaining sonicated material was added to the pre-bound antibody/protein G beads to rotate overnight at 4C. The next day the supernatant was discarded, and the beads were washed 5x with ice cold LiCl buffer (100mM Tris pH 7.5, 500mM LiCL, 1% NP40, 1% sodium deoxycholate) and 1x with ice cold TE buffer (10mM Tris pH 7.5, 1mM EDTA). Samples were eluted from the washed beads using room temperature IP elution buffer (1% SDS, 0.1 M $NaHCO_3$) at 65C for 1hr, vortexing every 15 minutes. The elution was then incubated at 65C overnight to reverse crosslinks. The next day DNA was purified using the QIAquick PCR purification kit (Qiagen 28104). DNA quantification was performed using the PicoGreen assay (Molecular Probes, Eugene, OR, P-7581). Sequencing libraries were generated from ChIP and input DNA by using the KAPA Hyper Prep kit (Roche, Basel, Switzerland, # 7962363001) according to the included manufacturer’s specifications, and quality was determined by using the Agilent TapeStation with D1000 screentape. Then, >50M 50-bp paired-end reads per sample were generated on the NovaSeq 6000. Reads were quality checked using fastqc (v0.11.5) and trimmed using trimgalore (v0.4.4) before being mapped to the hg19 reference genome using bowtie2 (v2.2.9). Sam files

were converted to bam format using samtools (v1.2), which were sorted using picard (v1.141). Duplicates were removed using picard and mitochondrial reads were removed using samtools. For visualization, bam files from replicates were merged using samtools and converted to bigwig format using deeptools (v3.5.0). For peak calling, we used macs2 (v2.1.1), and only considered peaks called in both samples. H3K27ac ChIP-seq data in Nalm6 cells was obtained from the Gene Expression Omnibus (GSE175484)[13].

PU.1 CUT and RUN

CUT and RUN data were generated using the Epicypher Cutana CUT&RUN kit v3.0 (14-1048) according to the manufacturers provided instructions. Briefly 500k NALM6 cells per reaction were bound to 10μl of provided activated ConA beads in 0.2ml PCR tubes. Bead-bound cells were suspended in Antibody Buffer (Wash buffer with 0.1% digitonin, 0.5mM Spermidine, 2mM EDTA, and 1x HALT protease inhibitors) and incubated with 1ul PU.1 antibody (Cell Signaling 2258) or IgG (Epicypher 13-0042k) overnight on a nutator mixer at 4C. The next day after washing, pAG MNase was bound and targeted digestion was carried out for 2 hours at 4C. Digestion was stopped using 33μl Stop buffer + 1μl (0.5ng) E.coli spike-in DNA and then cleaved DNA were released for 10 minutes at 37C. DNA were then purified for library preparation using the included purification kit. >30M 75bp paired end reads were generated per sample using the Illumina Novaseq. The Nextflow CUT and RUN pipeline (v2.0) was used in spike-in mode to assess quality and map reads to the HG19 (human) and K12-MG1655 (E. coli) reference genomes [14,15]. The spike-in normalized .bam files from Nextflow were exported to Easeq (v1.111), where peaks were called against the IgG sample using adaptive local thresholding ($p < 1x10^{-5}$, FDR $< 1x10^{-5}$, Log_2(Fold Change) > 1, merge within = 100bp, window size = 100bp) [16]. Data shown are spike-in normalized bigwig files generated in Nextflow.

CRISPR/Cas9 deletion and allele swaping

Regional deletions and *rs1247117* A>G modifications in Nalm6 and/or SUPB15 cells were generated using CRISPR-Cas9 technology. In brief, one million cells were transiently transfected with precomplexed ribonuclear proteins (RNPs) consisting of 100pmol of each chemically modified sgRNA (Synthego, see **Supplementary Data 14**), 35pmol of Cas9 protein (St. Jude Protein Production Core), and 3ug of ssODN (Alt-R modifications, IDT, see **Supplementary Data 14**) via nucleofection (Lonza, 4D-Nucleofector™ X-unit) using solution P3 and program CV-104 in a large (100ul) cuvette according to the manufacturer's recommended protocol. Three days post-nucleofection, genomic DNA was harvested via crude lysis and used for PCR amplification (see Sup File 3 for primers). The presence of the desired deletion was confirmed via gel electrophoresis and/or sequencing. To validate disruptions, targeted amplicons were generated using gene specific primers with partial Illumina adapter overhangs and sequenced. Cell pellets of approximately 10,000 cells were lysed and used to generate gene specific amplicons with partial Illumina adapters in PCR#1. Amplicons were indexed in PCR#2 and pooled with targeted amplicons from other loci to create sequence diversity. Additionally, 10% PhiX Sequencing Control V3 (Illumina) was added to the pooled amplicon library prior to running the sample on an Miseq Sequencer System (Illumina) to generate paired 2 X 250bp reads. Samples were demultiplexed using the index sequences, fastq files were generated, and NGS analysis was performed using CRIS.py [17].

Drug sensitivity assays

Parental cells (Nalm6 WT, and SUP-B15 WT), CRE deletion cells (Nalm6 Del, and SUP-B15 Del), Nalm6 WT (control), and Nalm6 EIF3A overexpression cells were seeded at 20,000 cells per well in a 96-well plate and co-treated with the indicated concentrations of vincristine (Hospira, 61703-0309-16), Mercaptopurine (6-MP), or Dexamethasone (Dex). Following the indicated duration of incubation, cell viability was measured using the CellTiter-Glo® 2.0 Cell Viability Assay (Promega,

G9243). The luminescence was measured using a BioTek Cytation1 cell imaging multimode reader (Agilent). The obtained values were normalized and plotted as % of vehicle treated cells. All the experiments were performed in 3 biological replicates of 3-6 technical replicates. Data was plotted as Mean +/− SD.

Caspase assay

Caspase assay in Nalm6 and *rs1247117* deletion cells was performed using a Caspase-Glo 3/7 assay kit (Promega, #G8091). This assay measures caspase-3 and -7 activities by inducing cell lysis, following by cleavage of the substrate, and generating luminescent signal. Briefly, 20,000 cells/well from each group were seeded in a 96-well plate and co-treated with vehicle or vincristine. After 72h of incubation, cells were mixed with the Caspase-Glo 3/7 reagent and measured the luminescence in a plate-reading luminometer (BioTek Cytation1, Agilent) following the manufacturer's instructions.

Western blotting

Whole cell lysates were harvested in RIPA buffer (Thermo Fisher Scientific, J63306.AP) for 20 minutes on ice, vortexing every 3 minutes. Supernatant lysates were quantified using Bradford's reagent (BioRad #5000205) and run on NuPAGE 12-4% Bis Tris gels and transferred overnight onto nitrocellulose membranes before blotting with the indicated antibodies. (ARHGAP18 abcam ab106553, HLTF abcam ab183042, TM4SF1 abcam ab113504, COMMD2 MilliporeSigma HPA044190, EIF3A Cell Signaling #3411, GYG1 abcam ab272606, β-Actin Cell Signaling #3700, Donkey Anti-Mouse IgG (H+L) Jackson Immunoresearch 615-035-214, Donkey Anti-Rabbit IgG (H+L) Jackson Immunoresearch 711-035-152)

Lentiviral overexpression of EIF3A in Nalm6 cells

Lentiviral control and *EIF3A* overexpression constructs and lentivirus were purchased from Vectorbuilder (VB010000-9298rtf & VB230824-1317ctk). For the infection of Nalm6 cells, 100k cells were spinfected in a 96-well plate for 2hr at 33°C at 1000xG in RPMI 1640 + 1% Glutamax + 10% fetal bovine serum + 8μg/ML polybrene at an MOI of 10. Immediately after spinfection the supernatant was discarded and replaced with fresh RPMI 1640 + 1% Glutamax + 10% fetal bovine serum. After 72hrs, we initiated selection of infected cells with RPMI 1640 + 1% Glutamax + 10% fetal bovine serum + 0.5μg/ML puromycin for 7 days before beginning experiments.

Antibodies

- Rabbit PU.1 antibody (9G7) (#2258S, Cell Signaling Technology, lot 5) (CUT & RUN (1:50), ChIP (1:20), and western blotting (1:3000))
- Mouse RNA polymerase II CTD repeat YSPTSPS (phospho S5) antibody [4H8] (ab5408, ABCAM lot: GR3264797-1) (CHIP (5μg))
- Anti-Glycogenin 1 antibody (ab272606 lot: 1006756-2) (western blotting (1:1000))
- Anti-HLTF antibody [EPR14761] (ab183042 lot: 1023533-2) (western blotting (1:1000))
- EIF3A (D51F4) XP® Rabbit mAb (#3411, Cell Signaling, lot 3) (western blotting (1:1000))
- β-Actin (8H10D10) Mouse mAb (#3700 Cell Signaling, lot 17) (western blotting (1:5000))
- Anti-COMMD2 antibody produced in rabbit (millipore sigma HPA044190 lot: r42272) (western blotting (1:1000))
- Anti-Transmembrane 4 L6 family member 1 antibody (abcam ab113504 lot: 1050893-6) (western blotting (1:1000))
- Anti-ARHGAP18 antibody (abcam ab106553 lot:1040959-3) (western blotting (1:1000))
- Anti H3K27Ac antibody (Active Motif 91194) (HiCHIP (0.2μg per 1μg of sheared chromatin))

Antibody validation

Rabbit PU.1 antibody (#2258S, Cell Signaling Technology) (CUT & RUN and western blotting) was validated by manufacturer and cited by other work. Please see: PU.1 https://www.cellsignal.com/products/primary-antibodies/pu-1-9g7-rabbit-mab/2258

Mouse RNA polymerase II CTD repeat YSPTSPS (phospho S5) antibody [4H8] (ab5408, ABCAM lot: GR3264797-1) (CHIP) was validated by manufacturer and cited by other work. Please see: https://www.abcam.com/rna-polymerase-ii-ctd-repeat-ysptsps-phospho-s5-antibody-4h8-chip-grade-ab5408.html

Anti-Glycogenin 1 antibody (abcam ab272606 lot: 1006756-2) (western blotting) detects band at the predicted size of GYG1 both by manufacturer and our work.

Anti-HLTF antibody [EPR14761] (abcam ab183042 lot: 1023533-2) (western blotting) was validated by manufacturer using knock-out and parental cells.

EIF3A (D51F4) XP® Rabbit mAb #3411 was validated by manufacturer and cited by other work. Please see: https://www.cellsignal.com/products/primary-antibodies/eif3a-d51f4-xp-174-rabbit-mab/3411

β-Actin (8H10D10) Mouse mAb #3700 was validated by manufacturer and cited by other work. Please see: https://www.cellsignal.com/products/primary-antibodies/b-actin-8h10d10-mouse-mab/3700

Anti-COMMD2 antibody produced in rabbit (millipore sigma HPA044190 lot: r42272) was validated by the human protein atlas and by the manufacturer. Please see: https://www.sigmaaldrich.com/US/en/product/sigma/hpa044190

Anti-Transmembrane 4 L6 family member 1 antibody (abcam ab113504 lot: 1050893-6) was validated by the manufacturer.

Anti-ARHGAP18 antibody (abcam ab106553 lot:1040959-3) was validated by the manufacturer and cited by other work. Please see: https://www.abcam.com/products/primary-antibodies/arhgap18-antibody-ab106553.html

Anti H3K27Ac antibody (Active Motif 91194) (HiCHIP) was validated by manufacturer for numerous functional genomic applications. Please see: https://www.activemotif.com/catalog/details/91193/abflex-histone-h3k27ac-antibody-rab

Statistical analysis

ATAC-QC Analysis

ATAC-seq samples from cell lines, frozen patient samples or fresh patient samples were assessed for data quality using the Encode Project defined transcription start site (TSS) enrichment score. Briefly, ATAC-seq sequencing read bam files mapped to the hg19 genome were downsized to chromosome 4 for faster quality assessment. TSS enrichment scores for each chromosome 4 bam file were computed using the ATACseqQC R-package (v. 1.26.0) and R programming language (v. 4.3.1) with command "TSSEscore(obj = Bam, txs = txdb, upstream = 2000, downstream = 2000)". TSS enrichment was computed using the TxDb.Hsapiens.UCSC.hg19.knownGene transcript database R-package (v. 3.2.2). Reproducibility of ATAC peaks was determined by defining

the unique set of peak summits within each sample category (cell line, frozen patient samples, or fresh patient sample). Unique summit intervals were subsequently intersected with a concatenated set of narrowPeak ATAC peak intervals corresponding to each sample category using bedtools (v. 2.30.0) command “bedtools intersect -c –a unique_summits.bed -b narrowPeaks.bed”. The count column of bedtools intersect was then utilized to determine peak reproducibility as the number of patient sample regions overlapping summit regions.

HiChIP Analysis

H3K27Ac HiChIP was processed using the Dovetail Genomics pipeline documented at https://hichip.readthedocs.io/en/latest/index.html. In preparation for peak calling processed HiChIP bam files were transformed into bed files using samtools (v. 1.14) and bedtools (v. 2.30.0) with command “samtools view -h -@ $([18] -F 0x900 sorted.mapped.PT.bam | bedtools bamtobed -i stdin > primary.aln.bed”. Peak calling to define anchor regions of interest was performed using MACS2 (v. 2.1.1) with command “macs2 callpeak -t primary.aln.bed --nomodel --extsize 147 -q 0.05”. Final statistically significant loops were identified utilizing FitHiChIP (v. 11.0) with configuration parameters “IntType=3, BINSIZE=5000, UppDistThr=2000000, UseP2PBackgrnd=0, BiasType=1, MergeInt=1, QVALUE=0.05”.

PU.1 ChIP PCR

2-tailed Two-way ANOVA with Dunnett’s correction was used to determine if parental Nalm6 cells showed a difference in PU.1 binding at rs1247117 relative A.G correction clones.

PU.1 binding affinity assay

A one-tailed student's T-test was used to test the hypothesis that the alternate allele would show less affinity for PU.1 binding.

PU.1 CUT and RUN

PU.1 peaks were called against the IgG sample using adaptive local thresholding ($p < 1x10^{-5}$, FDR < $1x10^{-5}$, Log_2(Fold Change) > 1, merge within = 100bp, window size = 100bp) [16]. Data shown are spike-in normalized bigwig files generated in Nextflow.

Drug sensitivity assays

For viability and caspase activity assays non-linear regression F-test was used to test the hypothesis that the dose-response curves of parental and modified ALL cells treated with the indicated therapeutic agent were different. We display the LC50 for the control sample calculated from the curve generated from the data.

Comparisons between groups of variant MPRA data

When comparing promoter-associated and distal promoter-connected variant MPRA data, the Mann-Whitney test was used. Comparisons within the distal promoter-connected variants from introns, UTRs and distal intergenic regions were carried out using the Kruskal-Wallis test with Dunn's correction for multiple comparisons.

***EIF3A* qPCR and Dual Luciferase reporter assays**

Student's T tests were used to determine the significance of differences between samples.

Western Blotting

One sample T-tests comparing the experimental group or groups to the control group (value of 1) were used to assess differences between quantified western blot images.

References

1 Kheradpour, P. *et al.* Systematic dissection of regulatory motifs in 2000 predicted human enhancers using a massively parallel reporter assay. *Genome Res* **23**, 800-811 (2013). https://doi.org/10.1101/gr.144899.112

2 Melnikov, A. *et al.* Systematic dissection and optimization of inducible enhancers in human cells using a massively parallel reporter assay. *Nature biotechnology* **30**, 271-277 (2012).

3 Melnikov, A., Zhang, X., Rogov, P., Wang, L. & Mikkelsen, T. S. Massively parallel reporter assays in cultured mammalian cells. *JoVE (Journal of Visualized Experiments)*, e51719 (2014).

4 Choi, J. *et al.* Massively parallel reporter assays of melanoma risk variants identify MX2 as a gene promoting melanoma. *Nat Commun* **11**, 2718 (2020). https://doi.org/10.1038/s41467-020-16590-1

5 Myint, L., Avramopoulos, D. G., Goff, L. A. & Hansen, K. D. Linear models enable powerful differential activity analysis in massively parallel reporter assays. *BMC Genomics* **20**, 209 (2019). https://doi.org/10.1186/s12864-019-5556-x

6 Corces, M. R. *et al.* Lineage-specific and single-cell chromatin accessibility charts human hematopoiesis and leukemia evolution. *Nature Genetics* **48**, 1193-1203 (2016). https://doi.org/10.1038/ng.3646

7 Bergeron, B. P. *et al.* Epigenomic profiling of glucocorticoid responses identifies cis-regulatory disruptions impacting steroid resistance in childhood acute lymphoblastic leukemia. *Leukemia* **36**, 2374-2383 (2022).

8 Barnett, K. R. *et al.* Epigenomic mapping reveals distinct B cell acute lymphoblastic leukemia chromatin architectures and regulators. *Cell Genom* **3**, 100442 (2023). https://doi.org/10.1016/j.xgen.2023.100442

9 Bergeron, B. P. *et al.* Mutual antagonism between glucocorticoid and canonical Wnt signaling pathways in B-cell acute lymphoblastic leukemia. *Blood Adv* **7**, 4107-4111 (2023). https://doi.org/10.1182/bloodadvances.2022009498

10 Wingett, S. W. *et al.* HiCUP: pipeline for mapping and processing Hi-C data. *F1000Research* **4**, 1310 (2015). https://doi.org/10.12688/f1000research.7334.1

11 Cairns, J. *et al.* CHiCAGO: robust detection of DNA looping interactions in Capture Hi-C data. *Genome Biology* **17** (2016). https://doi.org/10.1186/s13059-016-0992-2

12 Makowski, M. M. *et al.* An interaction proteomics survey of transcription factor binding at recurrent TERT promoter mutations. *Proteomics* **16**, 417-426 (2016).

13 Bergeron, B. P. *et al.* Epigenomic profiling of glucocorticoid responses identifies cis-regulatory disruptions impacting steroid resistance in childhood acute lymphoblastic leukemia. *Leukemia* **36**, 2374-2383 (2022). https://doi.org/10.1038/s41375-022-01685-z
14 Ewels, P. A. *et al.* The nf-core framework for community-curated bioinformatics pipelines. *Nature Biotechnology* **38**, 276-278 (2020). https://doi.org/10.1038/s41587-020-0439-x
15 Ewels, P. P., Alexander; Fillinger, Sven; Patel, Harshil; Alneberg, Johannes; Wilm, Andreas; Garcia, Maxime Ulysse; Di Tommaso, Paolo; Nahnsen, Sven. The nf-core framework for community-curated bioinformatics pipelines. *Zenodo* (2022). https://doi.org/10.5281/zenodo.7257061
16 Lerdrup, M., Johansen, J. V., Agrawal-Singh, S. & Hansen, K. An interactive environment for agile analysis and visualization of ChIP-sequencing data. *Nature Structural & Molecular Biology* **23**, 349-357 (2016). https://doi.org/10.1038/nsmb.3180
17 Connelly, J. P. & Pruett-Miller, S. M. CRIS.py: A Versatile and High-throughput Analysis Program for CRISPR-based Genome Editing. *Sci Rep* **9**, 4194 (2019). https://doi.org/10.1038/s41598-019-40896-w
18 Consortium, G. T. *et al.* Genetic effects on gene expression across human tissues. *Nature* **550**, 204-213 (2017). https://doi.org/10.1038/nature24277
