# Supplementary material for: Investigation of inherited noncoding genetic variation impacting the pharmacogenomics of childhood acute lymphoblastic leukemia treatment
Source: Nat Commun. 2024 May 1;15:3681. doi: 10.1038/s41467-024-48124-4 (PMC11063049; doi:10.1038/s41467-024-48124-4)
Supplement: Supplementary file 3 — Description of Additional Supplementary Files [file 41467_2024_48124_MOESM3_ESM.docx]

**Description of Additional Supplementary Files**

File Name: Supplementary Data 1

Description: Single nucleotide variants found in open chromatin and associated with ALL phenotypes

File Name: Supplementary Data 2

Description: Massively parallel reporter assay data

File Name: Supplementary Data 3

Description: Significant patient derived xenograft MPRA hits

File Name: Supplementary Data 4

Description: List of 556 reproducible and concordant regulatory variants from MPRA

File Name: Supplementary Data 5

Description: GREAT analysis of 556 reproducible and concordant regulatory variants

File Name: Supplementary Data 6

Description: Direct association of 54 reproducible and concordant regulatory variants with genes

File Name: Supplementary Data 7

Description: Significant chromatin loop interactions identified by H3K27Ac HiCHIP and promoter ChIC

File Name: Supplementary Data 8

Description: Sequences used for dual-luciferase reporter assays

File Name: Supplementary Data 9

Description: MPRA barcode sequences

File Name: Supplementary Data 10

Description: MPRA sample information and parameters

File Name: Supplementary Data 11

Description: MPRA oligo sequences

File Name: Supplementary Data 12

Description: rs1247117 PU.1 ChIP-qPCR primers

File Name: Supplementary Data 13

Description: PU.1 DNA pulldown oligo sequences

File Name: Supplementary Data 14

Description: CRISPR/Cas9 oligo sequences and validation
