# Supplementary material for: Investigation of inherited noncoding genetic variation impacting the pharmacogenomics of childhood acute lymphoblastic leukemia treatment
Source: Nat Commun. 2024 May 1;15:3681. doi: 10.1038/s41467-024-48124-4 (PMC11063049; doi:10.1038/s41467-024-48124-4)
Supplement: Supplementary file 18 — Reporting Summary [file 41467_2024_48124_MOESM18_ESM.pdf]

nature portfolio

Corresponding author(s): Daniel Savic

Last updated by author(s): Mar 8, 2024

Reporting Summary

Nature Portfolio wishes to improve the reproducibility of the work that we publish. This form provides structure for consistency and transparency in reporting. For further information on Nature Portfolio policies, see our Editorial Policies and the Editorial Policy Checklist.

Statistics

For all statistical analyses, confirm that the following items are present in the figure legend, table legend, main text, or Methods section.

n/a	Confirmed	
☐	☒	The exact sample size (*n*) for each experimental group/condition, given as a discrete number and unit of measurement
☐	☒	A statement on whether measurements were taken from distinct samples or whether the same sample was measured repeatedly
☐	☒	The statistical test(s) used AND whether they are one- or two-sided<br>*Only common tests should be described solely by name; describe more complex techniques in the Methods section.*
☒	☐	A description of all covariates tested
☐	☒	A description of any assumptions or corrections, such as tests of normality and adjustment for multiple comparisons
☐	☒	A full description of the statistical parameters including central tendency (e.g. means) or other basic estimates (e.g. regression coefficient) AND variation (e.g. standard deviation) or associated estimates of uncertainty (e.g. confidence intervals)
☐	☒	For null hypothesis testing, the test statistic (e.g. *F*, *t*, *r*) with confidence intervals, effect sizes, degrees of freedom and *P* value noted<br>*Give P values as exact values whenever suitable.*
☒	☐	For Bayesian analysis, information on the choice of priors and Markov chain Monte Carlo settings
☒	☐	For hierarchical and complex designs, identification of the appropriate level for tests and full reporting of outcomes
☒	☐	Estimates of effect sizes (e.g. Cohen's *d*, Pearson's *r*), indicating how they were calculated

Our web collection on statistics for biologists contains articles on many of the points above.

Software and code

Policy information about availability of computer code

Data collection	For sequencing (illumina), plate reading (Biotek (now agilent)), and blot/gel imaging (BioRad,Li-cor) the manufacturers included proprietary software for the indicated machine was used collect data.
Data analysis	-Graphpad Prism 9 was used to analyze and/or visualize data in Figures 1C; 2B-C; 3A-D; 4A-E; 5D,F-G,H; SF3A-K; SF4C-D.<br>-R base v4.4.2 programming was used to analyze and visualize data in Figure 2D with packages ggplot2 v3.4.0 and tidyverse v1.3.2. Please see the attached figure2d_data_visualization_and_stats.Rmd file.<br>-Integrative genomics viewer (IGV, v2.16.0) was used to generate Figures 5A,E; SF4A.<br>-PU.1 CUT&RUN data were initially processed using nextflow v21.10.6 nf-core/cutandrun: 2.0.0 (please see attached cut&run_software_versions.yml reporting file for all software used with nextflow). We exported spike-in normalized .bam files for IgG and PU.1 antibodies and called PU.1 peaks against the IgG using EASEQ using the parameters listed in the methods section (Easeq is freely available at https://easeq.net/).<br>-Promoter Capture HiC data was analyzed using the Arima Capture HiC Pipeline available at https://github.com/ArimaGenomics/CHiC<br>-HiChIP was analyzed using FithiChiP. https://github.com/ay-lab/FitHiChIP

For manuscripts utilizing custom algorithms or software that are central to the research but not yet described in published literature, software must be made available to editors and reviewers. We strongly encourage code deposition in a community repository (e.g. GitHub). See the Nature Portfolio guidelines for submitting code & software for further information.

Data

Policy information about availability of data

All manuscripts must include a data availability statement. This statement should provide the following information, where applicable:

- Accession codes, unique identifiers, or web links for publicly available datasets
- A description of any restrictions on data availability
- For clinical datasets or third party data, please ensure that the statement adheres to our policy

The ATAC-seq, Promoter capture HiC, HiChIP, RNA pol II, and PU.1 genomic binding data data generated in this study have been deposited in the GEO database under accession code GSE 224204 [GSE224204]. The fresh patient sample ATAC-seq data used in this study are available in the GEO database under accession code GSE226400 [GSE226400]. The frozen patient sample ATAC-seq data used in this study are available in the GEO database under accession code GSE161501 [GSE161501]. H3K27Ac ChIP-seq data, “GSE175482 Nalm6 H3K27ac 0hr merged.bw”, used in this study are available in the GEO database under accession code GSE175482 [GSE175482]. All other data generated in this study are provided in the Supplementary Information/Source Data file.

Human research participants

Policy information about studies involving human research participants and Sex and Gender in Research.

Reporting on sex and gender	Please see the published information for St. Jude Children’s Research Hospital (Memphis, Tennessee) Total Therapy XV (TOTXV, NCT00137111), Total Therapy XVI protocol (TOTXVI, NCT00549848) and Total Therapy XVII protocol (TOT17, NCT03117751) at:<br>https://clinicaltrials.gov/study/NCT00137111<br>https://clinicaltrials.gov/ct2/show/study/NCT00549848<br>https://clinicaltrials.gov/ct2/show/NCT03117751
Population characteristics	Ages 18 months to 18 years were included. Please see the published information for St. Jude Children’s Research Hospital (Memphis, Tennessee) Total Therapy XV (TOTXV, NCT00137111), Total Therapy XVI protocol (TOTXVI, NCT00549848) and Total Therapy XVII protocol (TOT17, NCT03117751) at:<br>https://clinicaltrials.gov/study/NCT00137111<br>https://clinicaltrials.gov/ct2/show/study/NCT00549848<br>https://clinicaltrials.gov/ct2/show/NCT03117751
Recruitment	Please see the published information for St. Jude Children’s Research Hospital (Memphis, Tennessee) Total Therapy XV (TOTXV, NCT00137111), Total Therapy XVI protocol (TOTXVI, NCT00549848) and Total Therapy XVII protocol (TOT17, NCT03117751) at:<br>https://clinicaltrials.gov/study/NCT00137111<br>https://clinicaltrials.gov/ct2/show/study/NCT00549848<br>https://clinicaltrials.gov/ct2/show/NCT03117751
Ethics oversight	The use of these samples was approved by the institutional review board at St. Jude Children’s Research Hospital.

Note that full information on the approval of the study protocol must also be provided in the manuscript.

Field-specific reporting

Please select the one below that is the best fit for your research. If you are not sure, read the appropriate sections before making your selection.

☒ Life sciences ☐ Behavioural & social sciences ☐ Ecological, evolutionary & environmental sciences

For a reference copy of the document with all sections, see nature.com/documents/nr-reporting-summary-flat.pdf

Life sciences study design

All studies must disclose on these points even when the disclosure is negative.

Sample size	-No statistical means of sample size calculation was used for this work<br>-For ALL chromatin accessibility mapping, sample size was determined by the patient samples and PDXs that were made available to us. For ALL cell lines, we used previously published T-ALL cell line data (7 cell lines) and decided to match those 7 with an additional 7 B-ALL cell lines of new data. These were performed in duplicate for each cell line.<br>-For MPRA cell lines we sampled 7 B-ALL and 3 T-ALL cell lines because B-ALL is more common than T-ALL, and because MPRA is a labor and cost intensive experiment. These were performed as 4 independent transfections per cell line.<br>-For promoter capture HiC and Hi ChIP we sampled 7 B-ALL cell lines and 1 T-ALL cell line cell lines because B-ALL is more common than T-ALL, and because these are labor and cost intensive experiments and we had confidently identified the interactions.<br>-For NALM6 Vincristine sensitivity assays we sampled 3 independent platings of cells per condition where there were 4-6 technical replicates per dose to ensure reproducibility and perform valid statistical analysis. These parameters allowed for sufficient sampling of the variability

that is expected in cell culture based experiments.
-For qPCR experiments 3 independent experiments were performed for each gene to account for variability associated with cell culture based experiments.
- For western blotting quantification, at least three independent experiments were used for statistics because that is the minimum required for the proper assessment of variance.
-For dual-luciferase assays 2-3 independent experiments were performed per variant. If a significant difference was observed with two replicates then two replicates were deemed sufficient. If not, we performed a third replicate.
-PU.1 in vitro pull downs were performed twice. After the experiment robustly resulted in the same answer twice, and we had orthogonal assays confirming the result we were satisfied.
- For PU.1 ChIP PCR, we performed three independent experiments so that variance could properly be assessed. We found that three replicates were appropriate given the robust nature of the experiment using three sets of primers and two independent clones, as well as extensive orthogonal data supporting the same conclusion.
-For ATAC-seq rs1247117 allele counting, we performed three independent experiments so that variance could properly be assessed. We found that three replicates were appropriate given the robust nature of the experiment using two independent clones, as well as extensive orthogonal data supporting the same conclusion.

Data exclusions

No data was excluded

Replication

-For cell culture-based experiments, independent experiments were performed independent cultures of the same cells. Technical replicates were considered samples taken from the same culture of cells. All technical replicates were taken at the same time, whereas independent experiments may or may not have been performed on the same day.
All experiments were reproducible with the exceptions listed below:
-Patient samples were not sampled a second time, therefore we have 2 technical replicates of the same sample for each patient ATAC-seq data, and pharmacotyping is only performed in technical replicates from one sample.
-PU.1 CUT and RUN data were only generated once for NALM6. We found the peak we were expecting at the rs1247117 locus as predicted by the PU.1 motif at that locus and the in vitro pull down experiments.

Randomization

Samples were assigned into groups randomly.

Blinding

Analyses were performed objectively and did not require blinding.

Reporting for specific materials, systems and methods

We require information from authors about some types of materials, experimental systems and methods used in many studies. Here, indicate whether each material, system or method listed is relevant to your study. If you are not sure if a list item applies to your research, read the appropriate section before selecting a response.

Materials & experimental systems

n/a	Involved in the study
☐	☒ Antibodies
☐	☒ Eukaryotic cell lines
☒	☐ Palaeontology and archaeology
☐	☒ Animals and other organisms
☒	☐ Clinical data
☒	☐ Dual use research of concern

Methods

n/a	Involved in the study
☐	☒ ChIP-seq
☒	☐ Flow cytometry
☒	☐ MRI-based neuroimaging

Antibodies

Antibodies used

rabbit PU.1 antibody (9G7) (#2258S, Cell Signaling Technology, lot 5) (CUT & RUN (1:50), ChIP (1:20), and western blotting (1:3000))
mouse RNA polymerase II CTD repeat YSPTSPS (phospho S5) antibody [4H8] (ab5408, ABCAM lot: GR3264797-1) (CHIP (5µg))
Anti-Glycogenin 1 antibody (ab272606 lot: 1006756-2) (western blotting (1:1000))
Anti-HLTF antibody [EPR14761] (ab183042 lot: 1023533-2) (western blotting (1:1000))
eIF3A (D51F4) XP® Rabbit mAb (#3411, Cell Signaling, lot 3) (western blotting (1:1000))
β-Actin (8H10D10) Mouse mAb (#3700 Cell Signaling, lot 17) (western blotting (1:5000))
Anti-COMMD2 antibody produced in rabbit (millipore sigma HPA044190 lot: r42272) (western blotting (1:1000))
Anti-Transmembrane 4 L6 family member 1 antibody (abcam ab113504 lot: 1050893-6) (western blotting (1:1000))
Anti-ARHGAP18 antibody (abcam ab106553 lot:1040959-3) (western blotting (1:1000))
Anti H3K27Ac antibody (Active Motif 91194) (HiCHIP (0.2µg per 1µg of sheared chromatin))

Validation

rabbit PU.1 antibody (#2258S, Cell Signaling Technology) (CUT & RUN and western blotting)-Validated by manufacturer and previous publications. Please see: PU.1 https://www.cellsignal.com/products/primary-antibodies/pu-1-9g7-rabbit-mab/2258 "This antibody

detects endogenous levels of total PU.1 protein. The antibody does not cross react with other Ets family members."

mouse RNA polymerase II CTD repeat YSPTSPS (phospho S5) antibody [4H8] (ab5408, ABCAM lot: GR3264797-1) (CHIP)-Validated by manufacturer and previous publications. Please see: https://www.abcam.com/rna-polymerase-ii-ctd-repeat-ysptsps-phospho-s5-antibody-4h8-chip-grade-ab5408.html "Abcam is leading the way in addressing this with our range of recombinant monoclonal antibodies and knockout edited cell lines for gold-standard validation."

Anti-Glycogenin 1 antibody (abcam ab272606 lot: 1006756-2) (western blotting) Detects band at the predicted size of GYG1 both by manufacturer and our work.

Anti-HLTF antibody [EPR14761] (abcam ab183042 lot: 1023533-2) (western blotting) Knock-out validated by manufacturer

eIF3A (D51F4) XP® Rabbit mAb #3411- validated by manufacturer and numerous citations (https://www.cellsignal.com/products/primary-antibodies/eif3a-d51f4-xp-174-rabbit-mab/3411)

β-Actin (8H10D10) Mouse mAb #3700- validated by manufacturer and numerous citations(https://www.cellsignal.com/products/primary-antibodies/b-actin-8h10d10-mouse-mab/3700)

Anti-COMMD2 antibody produced in rabbit (millipore sigma HPA044190 lot: r42272) validated by the human protein atlas and manufacturer (https://www.sigmaaldrich.com/US/en/product/sigma/hpa044190)

Anti-Transmembrane 4 L6 family member 1 antibody (abcam ab113504 lot: 1050893-6) validated by the manufacturer

Anti-ARHGAP18 antibody (abcam ab106553 lot:1040959-3) validated by the manufacturer and cited by other work. (https://www.abcam.com/products/primary-antibodies/arhgap18-antibody-ab106553.html)

Anti H3K27Ac antibody (Active Motif 91194) (HiCHIP) Validated by manufacturer in general use and Arima Genomics for HiChIP.

Eukaryotic cell lines

Policy information about cell lines and Sex and Gender in Research

Cell line source(s)	NALM6 DSMZ ACC128, Jurkat DSMZ ACC282, Ball-1 DSMZ ACC742, 697 DSMZ ACC42, CEM DSMZ ACC240, REH DSMZ ACC22, P12 DSMZ ACC34, SEM DSMZ ACC546, Loucy DSMZ ACC394, DND41 DSMZ ACC525, HSB2 DSMZ ACC435, MOLT16 DSMZ ACC29, RS411 DSMZ ACC508, SUPB15 DSMZ ACC389
Authentication	All cell lines were authenticated by DSMZ<br>We independently authenticated all cell lines by Powerplex STR profile.
Mycoplasma contamination	PCR-based mycoplasma testing determined that the cell lines were negative for mycoplasma contamination.
Commonly misidentified lines (See ICLAC register)	Cell lines used in this study were not found in the database of commonly misidentified lines (ICLAC).

Animals and other research organisms

Policy information about studies involving animals; ARRIVE guidelines recommended for reporting animal research, and Sex and Gender in Research

Laboratory animals	NOD.Cg-PrkdcscidIl2rgtm1Wjl/SzJ (NSG) (8-12 weeks of age) mice from Animal Resource Center at St. Jude
Wild animals	No wild animals

Reporting on sex	Sex was not considered for PDX studies. The animals used were female, however, cells harvested were human and used for MPRA where molecules harvested were artificial episomal RNA and DNA of neither human or mouse origin.
Field-collected samples	no field collection
Ethics oversight	The animal studies were approved by the Institutional Animal Care and Use Committee of St. Jude.

Note that full information on the approval of the study protocol must also be provided in the manuscript.

ChIP-seq

Data deposition

☒ Confirm that both raw and final processed data have been deposited in a public database such as GEO.

☒ Confirm that you have deposited or provided access to graph files (e.g. BED files) for the called peaks.

Data access links<br>*May remain private before publication.*	https://www.ncbi.nlm.nih.gov/geo/query/acc.cgi?acc=GSE224204
Files in database submission	2049027_Nalm6_input_PolII_ChIP_S52_L001_R1_001.fastq.gz<br>2049027_Nalm6_input_PolII_ChIP_S52_L001_R2_001.fastq.gz<br>2049027_Nalm6_input_PolII_ChIP_S52_L002_R1_001.fastq.gz<br>2049027_Nalm6_input_PolII_ChIP_S52_L002_R2_001.fastq.gz<br>2049017_Nalm6_0hr_Rep1_PolII_ChIP_S42_L001_R1_001.fastq.gz<br>2049017_Nalm6_0hr_Rep1_PolII_ChIP_S42_L001_R2_001.fastq.gz<br>2049017_Nalm6_0hr_Rep1_PolII_ChIP_S42_L002_R1_001.fastq.gz<br>2049017_Nalm6_0hr_Rep1_PolII_ChIP_S42_L002_R2_001.fastq.gz<br>2049018_Nalm6_0hr_Rep2_PolII_ChIP_S43_L001_R1_001.fastq.gz<br>2049018_Nalm6_0hr_Rep2_PolII_ChIP_S43_L001_R2_001.fastq.gz<br>2049018_Nalm6_0hr_Rep2_PolII_ChIP_S43_L002_R1_001.fastq.gz<br>2049018_Nalm6_0hr_Rep2_PolII_ChIP_S43_L002_R2_001.fastq.gz<br>2049017_Nalm6_0hr_Rep1_PolII_ChIP_peaks.narrowPeak<br>2049018_Nalm6_0hr_Rep2_PolII_ChIP_peaks.narrowPeak<br>Nalm6_RNAP2_0hr_rep1_rep2_merged.bw
Genome browser session<br>(e.g. UCSC)	These data are not yet public to be shared on a genome browser session, but bigwig and peak files are available to reviewers on GEO (using the reviewer token) for all genomic data so that they may view them on the genome browser of their choosing.

Methodology

Replicates	This is two technical replicates showing high reproducibility of RNA PolII peaks
Sequencing depth	RNA pol II samples are paired-end libraries with 50bp reads.<br>NALM6_PolII_rep1, input reads: 98259236, Unique reads: 68141042, peaks: 114893<br>NALM6_PolII_rep2, input reads: 103815153, Unique reads: 68420477, peaks: 127435<br>NALM6_input, input reads: NA , Unique reads: 56032828, peaks: NA
Antibodies	mouse RNA polymerase II CTD repeat YSPTSPS (phospho S5) antibody [4H8] (ab5408, ABCAM lot: GR3264797-1) (CHIP)
Peak calling parameters	MACS2 version 2.1.1<br># effective genome size = 2.70e+09<br># band width = 300<br># model fold = [5, 50]<br># qvalue cutoff = 5.00e-02<br># Larger dataset will be scaled towards smaller dataset.<br># Range for calculating regional lambda is: 1000 bps and 10000 bps<br># Broad region calling is off<br># Paired-End mode is off<br># MACS will save fragment pileup signal per million reads<br># control file = [2049027_Nalm6_input_PolII_NODUPS_NOMT.bam]<br># ChIP-seq file = [2049017_Nalm6_0hr_Rep1_PolII_ChIP_NODUPS_NOMT.bam]<br># ChIP-seq file = [2049018_Nalm6_0hr_Rep2_PolII_ChIP_NODUPS_NOMT.bam]
Data quality	PolII rep1 peaks over 5-fold enrichment: 33789/114893<br>PolII rep1 peaks FDR < 0.05 : all<br>PolII rep2 peaks over 5-fold enrichment: 40108/127435<br>PolII rep2 peaks FDR < 0.05: all<br>Peaks found in both samples: 81521
Software	Reads were quality checked using fastqc (v0.11.5) and trimmed using trimgalore (v0.4.4) before being mapped to the hg19 reference

Software

genome using bowtie2 (v2.2.9). Sam files were converted to bam format using samtools (v1.2), which were sorted using picard (v1.141). Duplicates were removed using picard and mitochondrial reads were removed using samtools. For visualization, bam files from replicates were merged using samtools and converted to bigwig format using deeptools (v3.5.0). For peak calling, we used macs2 (v2.1.1), and only considered peaks called in both samples. H3K27ac ChIP-seq data in Nalm6 cells was obtained from the Gene Expression Omnibus (GSE161501).
